# Next-generation sequencing of five new avian paramyxoviruses 8 isolates from Kazakhstan indicates a low genetic evolution rate over four decades

**DOI:** 10.1007/s00705-017-3593-9

**Published:** 2017-10-22

**Authors:** Sasan Fereidouni, M. Jenckel, A. Seidalina, K. Karamendin, M. Beer, E. Starick, S. Asanova, E. Kasymbekov, M. Sayatov, A. Kydyrmanov

**Affiliations:** 1 Research Institute of Wildlife Ecology, University of Veterinary Medicine Vienna, Vienna, Austria; 2 Friedrich Loeffler Institute, Insel Riems, Germany; 3 Laboratory of Viral Ecology, Institute of Microbiology and Virology, Almaty, Kazakhstan

## Abstract

Five avian paramyxoviruses of serotype 8 (APMV-8) were isolated during a study monitoring wild birds in Kazakhstan in 2013 and each was further characterized. The viruses were isolated from three White-fronted geese (*Anser albifrons*), one Whooper swan (*Cygnus cygnus*), and one Little stint (*Calidris minuta*). Before our study, only two complete APMV-8 sequences had been reported worldwide since their discovery in the USA and Japan in the 1970s. We report the complete genome sequences of the newly detected viruses and analyze the genetic evolution of the APMV-8 viruses over four decades.

## Introduction

Avian paramyxoviruses (APMVs) can be taxonomically classified within the genus *Avulavirus* of the family *Paramyxoviridae* and include 15 serotypes: APMV-1 through 15 [1, 6, 16, 19, 20]. APMV-1, or Newcastle disease virus (NDV), is the most frequently isolated APMV and is economically the most important viral disease for the poultry industry [1]. Therefore, extensive research has been conducted on APMV-1, whereas very little is known about the molecular and biological characteristics of the other serotypes. Apart from APMV-5, all APMV’s have been reported in wild birds, and several serotypes, including APMV-10, 11, 12, 13 and 14 have been isolated exclusively from wild birds [6]. Thus far, reports of APMV-4, 8, 9, 12 and 14 have been restricted to ducks and geese [6].

The first two strains of APMV-8 were isolated from a Canadian goose in the USA in 1976 and a pintail in Japan in 1978 [2, 26]. At that time, they were characterized and considered as a new serotype based on hemagglutination inhibition test (HI) and double immunodiffusion assay. The whole genome sequences of these strains were recently published in GenBank [15] and their complete sequences are available as APMV-8/goose/Delaware/1053/76 (considered as the prototype strain) and APMV-8/pintail/Wakuya/20/78 [NCBI, updated 1st Jun. 2017]. In spite of accounts identifying APMV-8 during wild bird monitoring studies as well as a reported 48% seroprevalence in commercially produced chickens in the United States [25], only one more APMV-8 sequence has since been reported: a short hemagglutinin-neuraminidase (HN) sequence from a Tundra swan in Japan [23]. The molecular understanding of this serotype is therefore limited.

In this study, we report the complete genome sequences of five new APMV-8 isolates from Kazakhstan and discuss their genetic relationship to one another and in comparison to the other available sequences.

## Materials & Methods

### Sampling and preliminary identification

During a study monitoring wild birds in 2013 [13], five hemagglutinating viruses were isolated from cloacal swab samples from three White-fronted geese, one Whooper swan and one Little stint by inoculation in 10-days-old embryonated chicken eggs [10]. Viral RNA was extracted from infected allantoic fluid using QIAamp Viral RNA Mini Kit (Qiagen, Germany) according to the manufacturer’s instructions. The pan-PMV primers targeting conserved motifs of the polymerase gene sequences of the paramyxovirus family were successfully applied [21], and the same primers were used for sequencing of a short genome fragment using an ABI 3130xl DNA analyzer (Applied Biosystems). The BLAST search confirmed the relatedness of the isolated viruses with APMV-8, and the different isolates were designated as APMV-8/white-fronted goose/Kazakhstan/62/2013, APMV-8/white-fronted goose/Kazakhstan/92/2013, APMV-8/white-fronted goose/Kazakhstan/65/2013, APMV-8/whooper swan/Kazakhstan/95/2013, APMV-8/ little stint/Kazakhstan/14/2013. The samples were negative for avian influenza viruses as tested by a duplex RT-PCR targeting the influenza virus matrix gene [9] and a generic real-time RT-PCR that amplifies a part of the NP gene [5]. Hemagglutination inhibition tests using reference sera specific to APMV-1 through APMV-9 (National Reference Laboratory for NDV, Friedrich-Loeffler-Institute, Insel Riems, Germany) confirmed that the newly detected viruses belong to the APMV-8 serotype.

### Sequencing and data analysis

For whole genome sequencing, viral RNA served as a template for double-stranded cDNA synthesis using the cDNA Synthesis System (Roche, Mannheim, Germany). In summary, RNA was hybridized with random hexanucleotides and subsequently, first strand and second strand syntheses were performed according to the manufacturer’s instructions (Roche, Mannheim, Germany). The Covaris M220 ultrasonicator was used for DNA fragmentation of 0.5-1 µg of double-stranded DNA to an average size of about 300 base-pairs. For library preparation of the fragmented DNA, Illumina adaptors (Biooscientific, Austin, USA) and SPRIworks Fragment Library Cartridge II (Beckman Coulter, USA) were used on a SPRI-TE library system (Beckman Coulter, Fullerton, USA) with manual size selection afterwards. Upper and lower size selection was performed using AMPure XP magnetic beads (Beckmann Coulter). The quality of the library was checked on a Bioanalyzer 2100 (Agilent Technologies, Böblingen, Germany) using a High Sensitivity DNA Chip and corresponding reagents. Quantity was determined via qPCR with Kapa Library Quantification Kit (Kapa Biosystems, Cape Town, South Africa). Paired-end sequencing was performed on an Illumina MiSeq using MiSeq reagent kit v3 (Illumina, San Diego, USA). Raw sequence data was analyzed and assembled using the Genome Sequencer software suite (v. 2.8, Roche).

### Pathogenicity test

The virulence of the APMV-8 isolates was assessed by inoculation of the virus into embryonated SPF eggs followed by calculation of the mean death time (MDT) of the embryos. In addition, the fusion protein cleavage site was evaluated after sequencing of the corresponding gene.

### Phylogenetic analyses

**Fig. 1:**
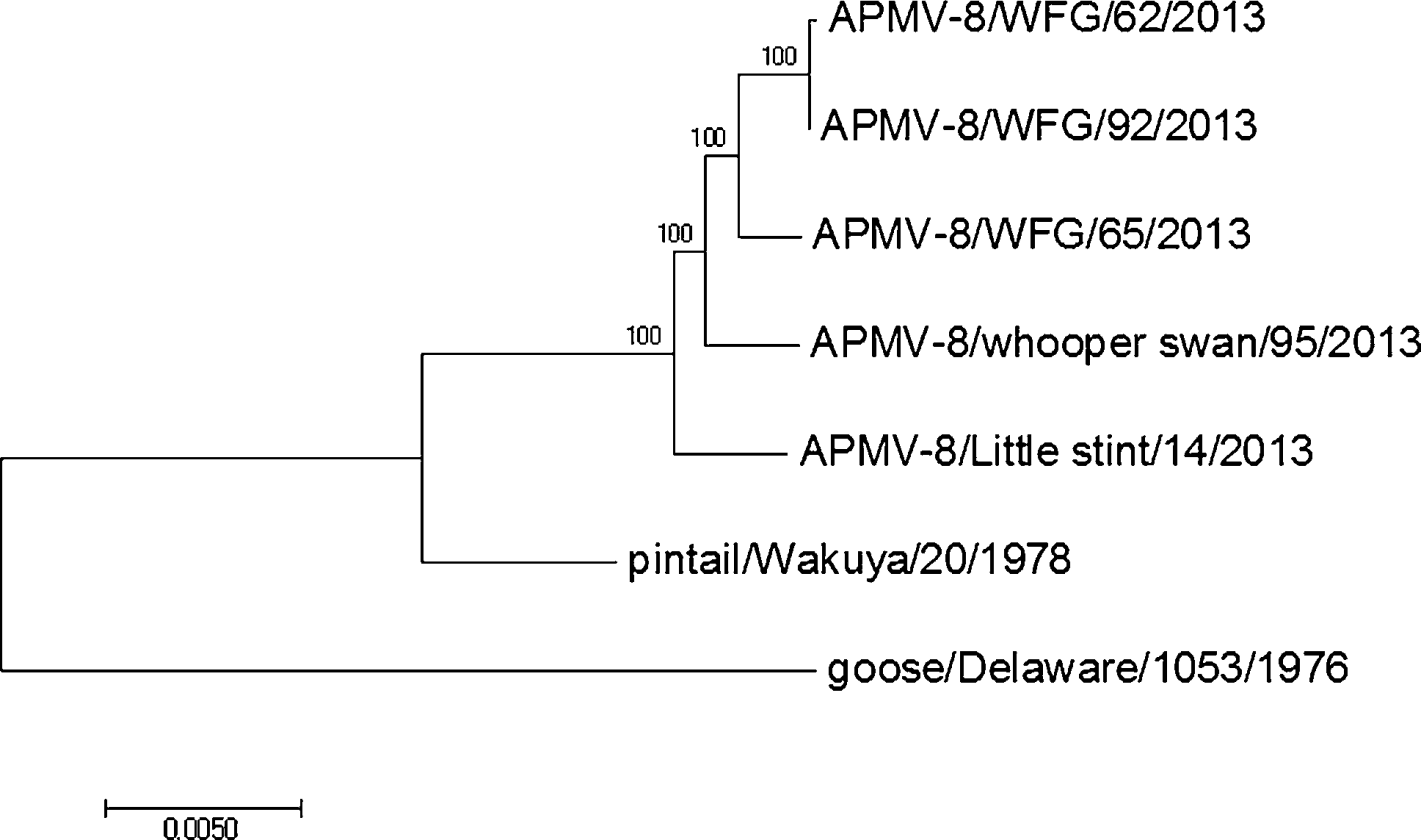
Phylogenetic analyses of the complete genome of five Kazakhstan APMV-8 strains and two previously reported isolates

Phylogenetic trees were generated on the basis of nucleotide (nt) and amino acid sequence alignments: a) separately for the six individual APMV genes (*N*, *P*, *M*, *F*, *HN and L*) and b) complete genomes (Fig. 1). The trees were generated using the maximum-likelihood approach (PAUP 4.0 and MEGA 7.0 software) based on the GTR + I+G model as suggested by the Akaike Information Criterion (AIC) and Bayesian Information Criterion (BIC) (ModelTest, embedded in TOPALi v2.5). The phylogenetic trees are rooted at the midpoint. The numbers at the nodes indicate maximum likelihood bootstrap values of 1000 replicates under the specified model. Only values above 0.85 are shown.

## Results

### Growth characteristics of APMV-8

Avian paramyxovirus type 8 strains from various wild birds isolated from Kazakhstan yielded hemagglutination (HA) titers of 32-128 units in the allantoic cavity of 10-day-old embryonated SPF chicken eggs and caused embryo death 3-4 days post-inoculation (Table 1).

**Table 1:** The hemagglutination titers and mean death time of APMV-8 isolates from Kazakhstan

Sequence ID	Strain	HA titer	Mean death time (hours)
WFG - 62	APMV-8/white-fronted goose/Kazakhstan/62/2013	32	70
WFG - 92	APMV-8/white-fronted goose/Kazakhstan/92/2013	32	94
WFG - 65	APMV-8/white-fronted goose/Kazakhstan/65/2013	128	94
Swan- 95	APMV-8/whooper swan/Kazakhstan/95/2013	64	94
Stint - 14	APMV-8/little stint /Kazakhstan/14/2013	128	70

### Determination of the complete genome sequence of the new APMV-8 strains

The sequence length of the five newly generated APMVs was identical to the two previously reported strains, goose/Delaware/1053/76 and pintail/Wakuya/20/78 (GenBank accession numbers FJ215863 and FJ215864, respectively): each of which consists of 15,342 nucleotides. In general, the genomes of APMV-8 strains (and APMV-1, -2, -3 and -4) consist of six tandemly linked genes in the order of *3′*- *Nucleoprotein* (*NP*) - *Phosphoprotein* (*P*) – *Matrix protein* (*M*) – *Fusion protein* (*F*) - *Hemagglutinin-neuraminidase* (*HN*) - *Large polymerase protein* (*L*) -*5′* yet lack the *SH* protein gene present in APMV-6 [10].

### Molecular analyses of the new APMV-8 strains

The five new isolates revealed several genetic differences amongst themselves. Two isolates, WFG-62 and WFG-92, showed the highest similarity, with only 3 nucleotide differences in the whole genome, but the other three viruses differed from 51 to 99 nucleotides (Table 2).

**Table 2:** Genetic difference among the APMV-8 isolates that were completely sequenced. The values indicate the number of nucleotide differences in whole genome between individual strains

	WFG - 62	WFG - 92	WFG - 65	Swan- 95	Stint - 14	Delaware
WFG - 92	3					
WFG - 65	54	51				
Swan- 95	79	76	74			
Stint - 14	99	96	93	90		
Wakuya	222	219	215	214	209	
Delaware	552	549	540	542	539	494

The extragenic 3`-leader and 5`-trailer regions contain the conserved promoter sequences [15]. Therefore, it is not surprising that the 3′-leader region (comprising 55 nucleotides) of the five isolates from Kazakhstan were identical to that of the Wakuya strain. However, the Delaware strain differed by a single nucleotide at position 52. Furthermore, the 5′-trailer region of the Kazakhstan strains showed no variability among the three White-fronted goose isolates but 1 and 3 nucleotide differences were observed when compared to the sequences of the Whooper swan and the Little stint strains, respectively.

The NP protein encoding gene of APMV-8 strains is 1383 nucleotides long, and encodes for 460 amino acids (Table 3). All Kazakhstan strains differed by one amino acid at position 441 (having an S) compared to the Wakuya and Delaware strains (which have an N and K, respectively). In addition, the two Kazakhstan strains isolated from a Whooper swan and a Little stint differed from each other by one amino acid at position 430 (P instead of S) and 432 (I instead of T), respectively (Table 4).

**Table 3:** Genetic differences among the APMV-8 isolates identified in wild birds in Kazakhstan. The overall mean distance for each protein sequence was calculated using ‘Compute Overall Mean Distance’ option embedded in Mega 7 software (Molecular Evolutionary Genetics Analysis version 7.0)

Gene	Length (nt)	Protein coding length (nt)	Overall mean distance (nt)
*Nucleoprotein*	1570	1383	17.6
*Phosphoprotein*	1374	1215	16.8
*Matrix* protein	1385	1107	16.4
*Fusion* protein	1830	1629	24.1
*Hemagglutinin-neuraminidase protein*	1994	1731	30.5
*Large* polymerase protein	6897	6714	86.7

**Table 4:**
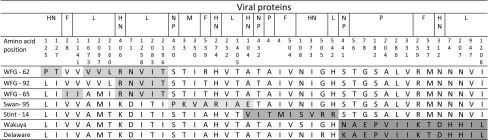
Amino acid residues located within six proteins of the Kazakhstan APMV8 isolates which can be used as genetic signatures to differentiate them from each other and previous sequenced isolates. The amino acid signatures (colored shadow) are grouped based on each viruses’ characteristics (instead of consecutive ordering) to be more informative, therefore viral proteins are consequently repeated

The P protein encoding gene is 1215 nucleotides long and encodes a protein of 404 amino acids. The Kazakhstan APMV-8 strains could be distinguished from the Wakuya and Delaware strains by 6 amino acid differences (Table 4); however, they differed from Wakuya and Delaware individually by 2 and 12 amino acids, respectively (Table 5).

**Table 5:**
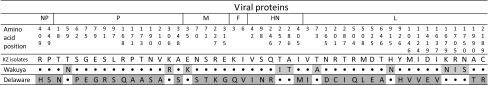
Amino acid residues located within six proteins of the Wakuya and Delaware isolates used as genetic signatures to differentiate them from each other and Kazakhstan isolates in general. The amino acid signatures are marked (colored shadow) based on consecutive ordering of the genes

The M protein encoding gene of the APMV-8 strains is 1107 nucleotides long and encodes a protein of 368 amino acids. The M-gene of APMV-8 is, in general, a conserved gene, and there are only seven amino acid differences among all strains (Tables 4, 5). Interestingly, two amino acids differentiate the swan strain from the other isolates (Table 4).

The F protein encoding gene of the APMV-8 strains is 1629 nucleotides long and encodes a proteins of 542 amino acids. The putative cleavage site of all available APMV-8 s, including the newly generated sequences, is T-Y-P-Q-T-R↓L (corresponding to amino acid positions 98-104) and this sequence does not show any variation. The cleavage site has only a single-basic residue, and therefore it does not indicate any potential for high pathogenicity.

The HN protein encoding gene of the APMV-8 isolates is 1731 nucleotides long and encodes a protein of 576 amino acids. The HN protein contains the hexapeptide N-R-K-S-C-S between amino acid positions 236 and 241, which is responsible for sialic acid binding at the cell surface [15]. The neuraminidase activity of the HN protein depends particularly on six amino acids (putative neuraminidase active site) at positions 176 (R), 401 (E), 416 (R), 506 (R), 534 (Y) and 555 (E) along with eleven conserved cysteine residues at amino acid positions 174, 188, 198, 240, 253, 346, 463, 469, 473, 539 and 550. These amino acids correspond to the globular head of the HN protein [15] and are identical for all identified APMV-8 isolates thus far.

The L protein encoding gene of the APMV-8 strains is 6714 nucleotides long and encodes a protein of 2238 amino acids. The L protein contains 20 amino acid variations that differentiate the strains Wakuya and Delaware, and it contains 15 additional amino acid variations that specify and differentiate various new isolates from Kazakhstan (Tables 4, 5).

### Genetic and phylogenetic analysis

The phylogenetic tree generated from the complete genome sequences perfectly reflect the trees for the single genes. Therefore, only the former tree was shown. The tree clearly indicate the very close genetic relationship between the different APMV-8 strains isolated from wild birds in Kazakhstan and show very few genetic variations (Fig. 1). The APMVs isolated from the White-fronted geese show a closer relationship among themselves when compared to the strains isolated from the Whooper swan and the Little stint. In addition, the APMV-8 isolated from the Whooper swan is more closely related to the strains from the White-fronted geese than to that of the Little stint.

## Discussion

Avian paramyxoviruses have been sporadically isolated from and identified in wild birds on different continents within the framework of sophisticated monitoring and surveillance studies [6]. Various paramyxoviruses have been detected with different incidence rates. APMV-8 and several other APMVs have rarely been characterized, and only two complete genome sequences were available in GenBank, APMV-8/goose/Delaware/1053/76 and APMV-8/pintail/Wakuya/20/78. Isolation and complete genome sequencing of more APMV-8 may therefore contribute to improving our knowledge of this subtype of paramyxoviruses.

The five newly isolated APMV-8 strains from Kazakhstan harbor the same genome size as the two previously reported completely sequenced isolates (15,342 nucleotides). Furthermore, the isolates from Kazakhstan showed a very close genetic relatedness amongst themselves, but they still displayed (with the exception of WFG-62 and WFG-92) differing levels of variability over their genes. All viruses were isolated from wild birds in 2013 but from different geographical regions. The three APMV-8 isolates from White-fronted geese were identified in wild birds sampled in a wetland in north of Kazakhstan and showed higher genetic relatedness when compared to isolates from a Whooper swan and a Little stint, respectively (Table 2 and Fig. 1). Whether this close genetic relationship is due to the fact that the birds belong to the same avian order or due to the fact that they were sampled in the same region and at the same time is unclear.

We also found very clear genetic signatures at the amino acid level, which allowed all viruses to be differentiated from each other (Tables 4, 5). Obviously, the number of differences at the nucleotide level is much higher (Table 2). The Central Asian isolates are more closely related to the East-Asian isolate (Wakuya) than to the North American (Delaware) ones. However, the time interval between the isolation of the Wakuya strain and APMV-8 viruses from Kazakhstan is more than 40 years. Although data from field surveillance studies and mortality reports did not indicate that APMV-8 strains are pathogenic for waterfowl, a positive selection due to the natural mutation of RNA viruses over time may be expected. The number of synonymous and non-synonymous mutations varied from gene to gene, and did not simply correlate to the gene size. In fact the number of amino acid substitutions per 100 amino acids showed great differences among the various proteins. The minimum and maximum substitutions belonged to the P and HN proteins, respectively (Tables 4, 5). High substitution rates for the surface proteins have been observed previously for other PMVs [17]. The putative F protein cleavage sites in the APMV-8 isolates from Kazakhstan were identical to the previously reported isolates, with the monobasic motif P-Q-T-R↓L sequence being present, and can therefore be considered as non-virulent APMV.

Interestingly, chickens immunized through the oculo-nasal route using the APMV-8 strain goose/Delaware/1053/76 that were later challenged by a virulent NDV showed reduced virus shedding via the respiratory system [14]. APMV-8/goose/Delaware/1053/76 also showed high cross-reactivity with sera from chickens previously infected with several APMV-1 strains. However, the cross-reactivity was much lower when a virus-neutralization test was used [22]. Most probably, antibodies against the F-protein of NDV contribute more to virus neutralization than antibodies against the HN protein [22]. The APMV-8 Delaware strain also showed replication in mice and hamsters, but failed to cause any infection in mallard ducks and displayed only limited replication in chicken [11, 12, 18].

Herein, we isolated APMV viruses from White-fronted geese, a Little stint and a Whooper swan, bird species that are non-target avian species and less commonly included in wild bird sampling studies in this region [4]. In addition, the newly detected positive samples originated from a geographic region that is an important breeding and staging area for migratory waterfowl in North Eurasia [24]. Conversely, no APMV-8 was found in a broad study of waders in Germany [8], or in shorebirds, gulls and waterfowl in the USA [3, 7]. In addition, since the initial global outbreaks of highly pathogenic avian influenza viruses in 2006, several hundred thousand samples from wild bird were collected and tested for avian influenza viruses. In many cases, those samples were also investigated for paramyxoviruses, but APMV-8 sequences have never been reported. This raises the question of whether the low number of isolated/identified APMV-8 might reflect a narrow host range or a confined geographical range of virus perpetuation. The seven reported APMV-8 sequences were identified in five different wild bird species mainly belonging to *Anseriformes*, the most representative birds in wild bird monitoring studies worldwide. Therefore, a narrow host range does not probably explain the rare identification of APMV-8 in wild birds. The geographical focus area of this study may provide the optimal conditions/environment for this subtype of paramyxoviruses, and this hypothesis should be evaluated further through additional wild bird monitoring studies in this region.

## Nucleotide sequence accession numbers

The complete genome sequences of the five APMV-8 strains (APMV-8/white-fronted goose/Kazakhstan/62/2013, APMV-8/white-fronted goose/Kazakhstan/92/2013, APMV-8/white-fronted goose/Kazakhstan/65/2013, APMV-8/whooper swan/Kazakhstan/95/2013, APMV-8/little stint /Kazakhstan/14/2013) are available at GenBank (accession numbers MF448511-MF448515, respectively).
